# 1-Methyl-3-(3-oxocyclo­hex-1-en­yl)azepan-2-one

**DOI:** 10.1107/S160053680802895X

**Published:** 2008-09-13

**Authors:** Wei-jia Liu, Hua-Jie Yuan, Lei Chen, Li Hai, Yong Wu

**Affiliations:** a Key Laboratory of Drug Targeting of the Education Ministry, West China School of Pharmacy, Sichuan University, Chengdu 610041, People’s Republic of China

## Abstract

The title compound, C_13_H_19_NO_2_, is a inter­mediate in the synthesis of the opioid analgesic meptazinol. In the crystal structure, a weak inter­molecular C—H⋯O inter­action occurs.

## Related literature

For related literature, see: Bradley *et al.* (1980 ▶); Hoskin & Hanks (1991 ▶).
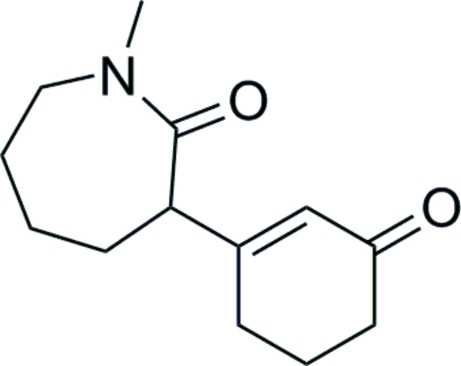


## Experimental

### 

#### Crystal data

C_13_H_19_NO_2_
*M* *_r_* = 221.29
Monoclinic, 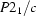

*a* = 9.450 (4) Å
*b* = 10.665 (3) Å
*c* = 11.963 (4) Å
β = 95.33 (3)°
*V* = 1200.5 (7) Å^3^
*Z* = 4
Mo *K*α radiation
μ = 0.08 mm^−1^
*T* = 294 (2) K
0.46 × 0.44 × 0.40 mm

#### Data collection

Enraf–Nonius CAD-4 diffractometer
Absorption correction: none
2361 measured reflections
2198 independent reflections
1235 reflections with *I* > 2σ(*I*)
*R*_int_ = 0.005
3 standard reflections every 150 reflections intensity decay: 0.7%

#### Refinement

*R*[*F* ^2^ > 2σ(*F* ^2^)] = 0.055
*wR*(*F* ^2^) = 0.163
*S* = 1.06
2198 reflections
146 parameters
H-atom parameters constrained
Δρ_max_ = 0.34 e Å^−3^
Δρ_min_ = −0.25 e Å^−3^

### 

Data collection: *DIFRAC* (Gabe & White, 1993 ▶); cell refinement: *DIFRAC*; data reduction: *NRCVAX* (Gabe *et al.*, 1989 ▶); program(s) used to solve structure: *SHELXS97* (Sheldrick, 2008 ▶); program(s) used to refine structure: *SHELXL97* (Sheldrick, 2008 ▶); molecular graphics: *ORTEP-3* (Farrugia, 1997 ▶); software used to prepare material for publication: *SHELXL97*.

## Supplementary Material

Crystal structure: contains datablocks global, I. DOI: 10.1107/S160053680802895X/hb2790sup1.cif

Structure factors: contains datablocks I. DOI: 10.1107/S160053680802895X/hb2790Isup2.hkl

Additional supplementary materials:  crystallographic information; 3D view; checkCIF report

## Figures and Tables

**Table 1:** Hydrogen-bond geometry (Å, °)

*D*—H⋯*A*	*D*—H	H⋯*A*	*D*⋯*A*	*D*—H⋯*A*
C7—H7*B*⋯O1^i^	0.96	2.54	3.436 (4)	155

Symmetry code: (i) 
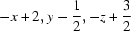
.

**Fig. 1.**
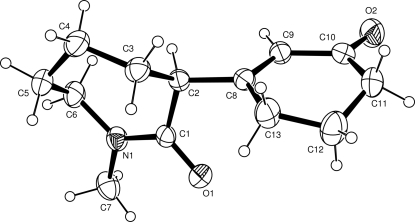
The molecular structure of (I), with displacement ellipsoids for the non-hydrogen atoms drawn at the 50% probability level.

## supplementary crystallographic information

### Comment

Meptazinol, 1-methyl-3-ethyl-3-(3-hydroxyphenyl)hexahydro-1*H*-azepin hydrochloride, is a synthetic hexahydroazepine derivative with opioid agonist and antagonist properties (Hoskin & Hanks, 1991). The title compound, (I) is a key intermediate for the synthesis of Meptazinol (Bradley *et al.*, 1980) and we report its structure here (Fig. 1).

The molecule of (I) is chiral. In the arbitrarily chosen asymmetric molecule, C2 has S configuration, but crystal symmetry generates a racemic mixture. In the crystal, a weak C—H···O interaction may help to consolidate the packing (Table 1).

### Experimental

A solution of butyl lithium (164 mmol) in hexane, maintained at 248 K was treated with diisopropylamine (13.5 ml, 164 mol) in THF (15 ml), followed by 1-methylazepan-2-one (8.1 g, 64 mmol) in THF (15 ml). After 10 min, a solution of 3-isopropoxy-2-cyclohexxenone (7.0 g, 45 mmol) in THF (10 ml) was added, the mixture allowed to warm to room temperature and after a further 2 h was acidified with 2 *M* hydrochloric acid. After 30 min, the aqueous layer was extracted with dichloromethane, the combined organic layer washed with brine and evaporated.  Recrystallization of the residue was from an ethyl acetate and hexane mixture. Colourless blocks of (I) were obtained by spontaneous evaporation in ethyl acetate and hexane (20:1 v/v).

### Refinement

The H atoms were positioned geometrically (C—H = 0.93–0.98 Å) and refined as riding with *U*_iso_(H) = 1.2*U*_eq_(C) or 1.5U_eq_(methyl C).

### Crystal data

**Table:**

C_13_H_19_NO_2_	*F*(000) = 480
*M**_r_* = 221.29	*D*_x_ = 1.224 Mg m^−^^3^
Monoclinic, *P*2_1_/*c*	Mo *K*α radiation, λ = 0.71073 Å
Hall symbol: -P 2ybc	Cell parameters from 20 reflections
*a* = 9.450 (4) Å	θ = 4.2–7.3°
*b* = 10.665 (3) Å	µ = 0.08 mm^−^^1^
*c* = 11.963 (4) Å	*T* = 294 K
β = 95.33 (3)°	Block, colourless
*V* = 1200.5 (7) Å^3^	0.46 × 0.44 × 0.40 mm
*Z* = 4	

### Data collection

**Table:**

Enraf–Nonius CAD-4 diffractometer	*R*_int_ = 0.005
Radiation source: fine-focus sealed tube	θ_max_ = 25.5°, θ_min_ = 2.2°
graphite	*h* = −11→11
ω/2θ scans	*k* = 0→12
2361 measured reflections	*l* = −4→14
2198 independent reflections	3 standard reflections every 150 reflections
1235 reflections with *I* > 2σ(*I*)	intensity decay: 0.7%

### Refinement

**Table:**

Refinement on *F*^2^	Primary atom site location: structure-invariant direct methods
Least-squares matrix: full	Secondary atom site location: difference Fourier map
*R*[*F*^2^ > 2σ(*F*^2^)] = 0.055	Hydrogen site location: inferred from neighbouring sites
*wR*(*F*^2^) = 0.163	H-atom parameters constrained
*S* = 1.06	*w* = 1/[σ^2^(*F*_o_^2^) + (0.0788*P*)^2^ + 0.1025*P*] where *P* = (*F*_o_^2^ + 2*F*_c_^2^)/3
2198 reflections	(Δ/σ)_max_ < 0.001
146 parameters	Δρ_max_ = 0.34 e Å^−^^3^
0 restraints	Δρ_min_ = −0.25 e Å^−^^3^

### Special details

**Table:**

Geometry. All e.s.d.'s (except the e.s.d. in the dihedral angle between two l.s. planes) are estimated using the full covariance matrix. The cell e.s.d.'s are taken into account individually in the estimation of e.s.d.'s in distances, angles and torsion angles; correlations between e.s.d.'s in cell parameters are only used when they are defined by crystal symmetry. An approximate (isotropic) treatment of cell e.s.d.'s is used for estimating e.s.d.'s involving l.s. planes.
Refinement. Refinement of *F*^2^ against ALL reflections. The weighted *R*-factor *wR* and goodness of fit *S* are based on *F*^2^, conventional *R*-factors *R* are based on *F*, with *F* set to zero for negative *F*^2^. The threshold expression of *F*^2^ > σ(*F*^2^) is used only for calculating *R*-factors(gt) *etc*. and is not relevant to the choice of reflections for refinement. *R*-factors based on *F*^2^ are statistically about twice as large as those based on *F*, and *R*- factors based on ALL data will be even larger.

### Fractional atomic coordinates and isotropic or equivalent isotropic displacement parameters (Å^2^)

**Table:**

	*x*	*y*	*z*	*U*_iso_*/*U*_eq_	
O1	0.95711 (19)	0.09365 (17)	0.84861 (15)	0.0577 (5)	
O2	0.5608 (2)	0.4437 (2)	0.8362 (2)	0.0810 (7)	
N1	0.8874 (2)	−0.0883 (2)	0.76476 (17)	0.0513 (6)	
C1	0.8668 (3)	0.0106 (2)	0.83106 (19)	0.0419 (6)	
C2	0.7256 (2)	0.0187 (2)	0.88309 (18)	0.0426 (6)	
H2	0.6495	0.0047	0.8230	0.051*	
C3	0.7118 (3)	−0.0828 (2)	0.9732 (2)	0.0539 (7)	
H3A	0.8030	−0.0930	1.0165	0.065*	
H3B	0.6442	−0.0546	1.0240	0.065*	
C4	0.6637 (3)	−0.2092 (3)	0.9246 (2)	0.0638 (8)	
H4A	0.6529	−0.2664	0.9862	0.077*	
H4B	0.5709	−0.1989	0.8837	0.077*	
C5	0.7628 (3)	−0.2687 (3)	0.8468 (2)	0.0659 (8)	
H5A	0.8541	−0.2834	0.8887	0.079*	
H5B	0.7243	−0.3495	0.8224	0.079*	
C6	0.7860 (3)	−0.1915 (3)	0.7444 (2)	0.0608 (8)	
H6A	0.8194	−0.2463	0.6877	0.073*	
H6B	0.6954	−0.1571	0.7142	0.073*	
C7	1.0154 (3)	−0.0913 (3)	0.7053 (2)	0.0669 (9)	
H7A	0.9931	−0.0626	0.6297	0.100*	
H7B	1.0509	−0.1756	0.7043	0.100*	
H7C	1.0863	−0.0377	0.7428	0.100*	
C8	0.7091 (2)	0.1503 (2)	0.9263 (2)	0.0435 (6)	
C9	0.6343 (3)	0.2358 (2)	0.8651 (2)	0.0477 (7)	
H9	0.5871	0.2107	0.7971	0.057*	
C10	0.6223 (3)	0.3659 (3)	0.8985 (2)	0.0553 (7)	
C11	0.6880 (3)	0.4006 (3)	1.0140 (3)	0.0702 (9)	
H11A	0.7247	0.4854	1.0115	0.084*	
H11B	0.6142	0.4004	1.0652	0.084*	
C12	0.8034 (4)	0.3173 (3)	1.0592 (3)	0.0766 (10)	
H12A	0.8193	0.3316	1.1395	0.092*	
H12B	0.8895	0.3418	1.0266	0.092*	
C13	0.7821 (3)	0.1819 (3)	1.0404 (2)	0.0631 (8)	
H13A	0.7258	0.1490	1.0975	0.076*	
H13B	0.8738	0.1404	1.0490	0.076*	

### Atomic displacement parameters (Å^2^)

**Table:**

	*U*^11^	*U*^22^	*U*^33^	*U*^12^	*U*^13^	*U*^23^
O1	0.0533 (11)	0.0494 (12)	0.0708 (12)	−0.0065 (9)	0.0074 (9)	−0.0010 (9)
O2	0.0766 (15)	0.0520 (14)	0.1098 (18)	0.0121 (11)	−0.0160 (13)	0.0039 (12)
N1	0.0595 (14)	0.0449 (14)	0.0509 (12)	0.0038 (11)	0.0122 (10)	−0.0052 (11)
C1	0.0468 (14)	0.0371 (14)	0.0404 (13)	0.0028 (12)	−0.0037 (11)	0.0059 (12)
C2	0.0436 (14)	0.0423 (16)	0.0398 (12)	0.0036 (11)	−0.0066 (10)	−0.0034 (12)
C3	0.0548 (16)	0.0517 (18)	0.0549 (16)	−0.0035 (13)	0.0043 (13)	0.0060 (13)
C4	0.0605 (17)	0.0525 (18)	0.0776 (19)	−0.0097 (15)	0.0017 (15)	0.0109 (16)
C5	0.0652 (18)	0.0398 (16)	0.091 (2)	−0.0055 (14)	−0.0008 (17)	−0.0046 (16)
C6	0.0668 (18)	0.0487 (17)	0.0656 (17)	0.0024 (14)	−0.0002 (14)	−0.0200 (15)
C7	0.080 (2)	0.061 (2)	0.0636 (17)	0.0157 (16)	0.0262 (16)	0.0046 (15)
C8	0.0421 (13)	0.0441 (16)	0.0440 (13)	0.0013 (12)	0.0018 (11)	−0.0048 (12)
C9	0.0455 (14)	0.0474 (16)	0.0487 (14)	0.0036 (13)	−0.0042 (11)	−0.0061 (13)
C10	0.0407 (14)	0.0518 (18)	0.0734 (19)	0.0042 (14)	0.0057 (13)	−0.0026 (16)
C11	0.075 (2)	0.0511 (19)	0.084 (2)	0.0028 (16)	0.0042 (17)	−0.0231 (17)
C12	0.097 (3)	0.067 (2)	0.0618 (19)	−0.0014 (19)	−0.0136 (17)	−0.0134 (17)
C13	0.0736 (19)	0.062 (2)	0.0505 (16)	0.0072 (16)	−0.0119 (14)	−0.0152 (14)

### Geometric parameters (Å, °)

**Table:**

O1—C1	1.234 (3)	C6—H6A	0.9700
O2—C10	1.225 (3)	C6—H6B	0.9700
N1—C1	1.344 (3)	C7—H7A	0.9600
N1—C7	1.459 (3)	C7—H7B	0.9600
N1—C6	1.465 (3)	C7—H7C	0.9600
C1—C2	1.527 (3)	C8—C9	1.330 (3)
C2—C8	1.509 (3)	C8—C13	1.509 (3)
C2—C3	1.542 (3)	C9—C10	1.452 (4)
C2—H2	0.9800	C9—H9	0.9300
C3—C4	1.520 (4)	C10—C11	1.507 (4)
C3—H3A	0.9700	C11—C12	1.470 (4)
C3—H3B	0.9700	C11—H11A	0.9700
C4—C5	1.520 (4)	C11—H11B	0.9700
C4—H4A	0.9700	C12—C13	1.473 (4)
C4—H4B	0.9700	C12—H12A	0.9700
C5—C6	1.509 (4)	C12—H12B	0.9700
C5—H5A	0.9700	C13—H13A	0.9700
C5—H5B	0.9700	C13—H13B	0.9700
			
C1—N1—C7	118.5 (2)	H6A—C6—H6B	107.6
C1—N1—C6	124.0 (2)	N1—C7—H7A	109.5
C7—N1—C6	117.5 (2)	N1—C7—H7B	109.5
O1—C1—N1	121.8 (2)	H7A—C7—H7B	109.5
O1—C1—C2	120.4 (2)	N1—C7—H7C	109.5
N1—C1—C2	117.7 (2)	H7A—C7—H7C	109.5
C8—C2—C1	108.3 (2)	H7B—C7—H7C	109.5
C8—C2—C3	113.3 (2)	C9—C8—C2	121.0 (2)
C1—C2—C3	112.3 (2)	C9—C8—C13	121.3 (2)
C8—C2—H2	107.6	C2—C8—C13	117.6 (2)
C1—C2—H2	107.6	C8—C9—C10	123.7 (2)
C3—C2—H2	107.6	C8—C9—H9	118.1
C4—C3—C2	113.4 (2)	C10—C9—H9	118.1
C4—C3—H3A	108.9	O2—C10—C9	121.7 (3)
C2—C3—H3A	108.9	O2—C10—C11	121.5 (3)
C4—C3—H3B	108.9	C9—C10—C11	116.8 (2)
C2—C3—H3B	108.9	C12—C11—C10	114.6 (2)
H3A—C3—H3B	107.7	C12—C11—H11A	108.6
C5—C4—C3	115.1 (2)	C10—C11—H11A	108.6
C5—C4—H4A	108.5	C12—C11—H11B	108.6
C3—C4—H4A	108.5	C10—C11—H11B	108.6
C5—C4—H4B	108.5	H11A—C11—H11B	107.6
C3—C4—H4B	108.5	C11—C12—C13	116.7 (3)
H4A—C4—H4B	107.5	C11—C12—H12A	108.1
C6—C5—C4	114.4 (2)	C13—C12—H12A	108.1
C6—C5—H5A	108.7	C11—C12—H12B	108.1
C4—C5—H5A	108.7	C13—C12—H12B	108.1
C6—C5—H5B	108.7	H12A—C12—H12B	107.3
C4—C5—H5B	108.7	C12—C13—C8	113.6 (2)
H5A—C5—H5B	107.6	C12—C13—H13A	108.8
N1—C6—C5	114.6 (2)	C8—C13—H13A	108.8
N1—C6—H6A	108.6	C12—C13—H13B	108.8
C5—C6—H6A	108.6	C8—C13—H13B	108.8
N1—C6—H6B	108.6	H13A—C13—H13B	107.7
C5—C6—H6B	108.6		
			
C7—N1—C1—O1	−5.0 (3)	C1—C2—C8—C9	96.5 (3)
C6—N1—C1—O1	177.8 (2)	C3—C2—C8—C9	−138.2 (2)
C7—N1—C1—C2	173.9 (2)	C1—C2—C8—C13	−82.6 (3)
C6—N1—C1—C2	−3.4 (3)	C3—C2—C8—C13	42.6 (3)
O1—C1—C2—C8	14.2 (3)	C2—C8—C9—C10	−175.7 (2)
N1—C1—C2—C8	−164.7 (2)	C13—C8—C9—C10	3.4 (4)
O1—C1—C2—C3	−111.7 (3)	C8—C9—C10—O2	174.7 (3)
N1—C1—C2—C3	69.4 (3)	C8—C9—C10—C11	−5.8 (4)
C8—C2—C3—C4	154.6 (2)	O2—C10—C11—C12	−156.1 (3)
C1—C2—C3—C4	−82.2 (3)	C9—C10—C11—C12	24.4 (4)
C2—C3—C4—C5	61.1 (3)	C10—C11—C12—C13	−41.6 (4)
C3—C4—C5—C6	−60.1 (3)	C11—C12—C13—C8	38.6 (4)
C1—N1—C6—C5	−64.5 (3)	C9—C8—C13—C12	−19.4 (4)
C7—N1—C6—C5	118.2 (3)	C2—C8—C13—C12	159.8 (3)
C4—C5—C6—N1	79.0 (3)		

### Hydrogen-bond geometry (Å, °)

**Table:**

*D*—H···*A*	*D*—H	H···*A*	*D*···*A*	*D*—H···*A*
C7—H7B···O1^i^	0.96	2.54	3.436 (4)	155

Symmetry codes: (i) −*x*+2, *y*−1/2, −*z*+3/2.
